# Psychiatric comorbidity of headache in a medical relief camp in a rural area

**DOI:** 10.4103/0019-5545.31583

**Published:** 2006

**Authors:** Himanshu Sharma, Savan Shah

**Affiliations:** * Assistant Professor and Head, Department of Psychiatry, P.S. Medical College and S.K. Hospital, Karamsad 388325, Anand, Gujarat; ** Consultant Psychiatrist

**Keywords:** Headache, migraine, psychiatric morbidity

## Abstract

**Background:**

Headache is one of the most common complaints seen by primary care physicians, but very few well-planned studies have been conducted to know its prevalence.

**Aim:**

To study the prevalence of headache and associated psychiatric morbidity.

**Methods:**

A medical relief camp was held in village Mavta (near Ratlam in Madhya Pradesh) in 2002. Of a total of 1350 registered subjects, 80 with primary complaints of headache were referred to our expert team of psychiatrists.

**Results:**

Sixty-nine subjects (86.25%) had psychiatric morbidity-mainly affective disorders (depression) and panic disorder, dysthymia, alcohol and nicotine dependence. Subjects with migraine and depression were mostly women with onset of symptoms at an early age. Subjects with less education; who were unmarried or had lost a spouse; those with a nuclear family; who were unemployed and those with a family history and past history of mental illness, were all susceptible to headache and depression.

**Conclusion:**

Disturbed sleep, free floating anxiety, sad mood, lack of pleasure, body ache and fatigue were the main presenting complaints along with headache.

## INTRODUCTION

Headache is a nearly universal phenomenon with a one-year prevalence of 90% and a life-time prevalence of 99%. Headache is one of the most common complaints seen by primary care physicians. In the United States of America, 9% of adults consult physicians for headache during a year, of which 83% resort to self-medication.^1^ Several studies have described headache as the main somatic presentation of depression in primary and general healthcare settings, along with tiredness and weakness, multiple aches and pains, dizziness, palpitations and sleep disturbances.^1^–^3^

Breslau *et al.*^4^ found that the estimated risk for major depression associated with prior migraine, adjusted for sex and education, was 3.2. The risk associated with prior depression was 3.1. A shared aetiology between migraine and depression is implicated. A history of migraine is associated with increased life-time rate of anxiety disorders, illicit drug abuse disorders, nicotine dependence and suicide attempts.^5^ Merikangas *et al.* also observed a strong association between migraine and depression, bipolar illness, anxiety and panic disorder.^6^

In the Indian setting too, subjects present to general practitioners with predominant complaints of migraine or migraine-type headache. But very few well-planned studies have been conducted to study the prevalence of headache and associated psychiatric morbidity. The present study was planned keeping the above facts in view.

## METHODS

A multispecialty medical relief camp was organized under the auspices of Pramukhswami Medical College and Shree Krishna Hospital and Medical Research Centre at Mavta near Ratlam (Madhya Pradesh) in 2002. Of a total of 1350 subjects registered in the camp, 80 patients were referred to our expert team of psychiatrists with primary complaints of headache. Of these, 69 patients (86.25%) were found to have a primary psychiatric diagnosis. These patients were examined for the nature, course, duration, type of headache as well as nature of associated complaints.

The statistics used were expressed as percentages.

## RESULTS

**Fig 1:**
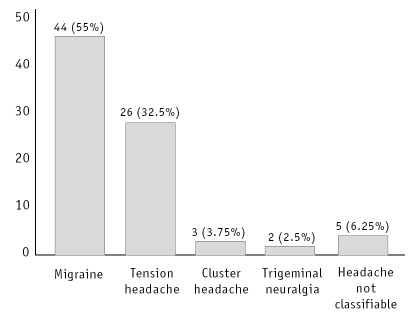
Break-up of neurological diagnosis

Figure 1 shows the break-up of neurological diagnosis of headache of the 80 patients according to the International Headache Society (IHS, 1988) classification.^7^ Migraine (with or without aura) was the most common complaint (44, 55%) followed by tension headache (26, 32.5%).

Sixty-nine out of 80 patients (86.25%) referred to us with complaints of headache had psychiatric morbidity according to the DSM-IV classification.^8^ Among these, 50 were men (72.5%) and 19 women. Their sociodemographic characteristics are given in Table 1. A majority of patients (72.5 %) were in the age group of 20–60 years, and 60 (86.5%) were earning less than Rs 2000 per month. Fifty-one subjects (74%) were literate and had received education up to Standard VIII and above. Family and past history of mental illness was present in 5 (7%) and 2 (3%) subjects, respectively. Substance dependence was present in 27 subjects (39%).

**Table 1:** Sociodemographic characteristics

		*n*	(%)
1.	Sex		
	Male	50	(72.5)
	Female	19	(27.5)
2.	Age		
	0–20 years	10	(14.5)
	20–40 years	20	(29.0)
	40–60 years	30	(43.5)
	60 years and above	9	(13.0)
3.	Average family income (Rs)		
	Less than 1000	27	(39.1)
	1000–2000	33	(47.8)
	more than 2000	9	(13.0)
4.	Education		
	Illiterate	18	(26.1)
	Up to Standard VIII	32	(46.4)
	Higher secondary	10	(14.5)
	Graduate	9	(13.0)
5.	Residence		
	Rural	69	(100)
	Urban	00	
6.	Religion		
	Hindu	64	(92.8)
	Muslim	5	(7.2)
7.	Marital status		
	Married	40	(58.0)
	Unmarried	23	(33.3)
	Lost a spouse	6	(8.7)
8.	Type of Family		
	Joint	41	(51.4)
	Nuclear	26	(37.7)
9.	Occupation		
	Service	10	(14.5)
	Self-employed	40	(58.0)
	Unemployed	19	(27.5)

Table 2 shows the break-up of psychiatric diagnosis (based on the DSM-IV classification): Major depressive disorder (MDD) in 22 (31.9%), panic disorder with agoraphobia in 10 (14.5%), phobia in 1 (2.5%), somatoform disorder in 4 (5.8%), substance dependence in 18 (26.1%), and dysthymia in 9 (13%).

**Table 2:** Break-up of psychiatric diagnosis (according to the DSM-IV classification)

S. No.	Diagnosis	*n*	(%)
1.	Major depressive disorder (MDD)	22	(31.9)
2.	Panic disorder with agoraphobia	10	(14.5)
3.	Phobia	2	(2.9)
4.	Somatoform disorder	4	(5.8)
5.	Substance dependence	18	(26.1)
6.	Dysthymia	9	(13.0)

Table 3 shows that complaints of headache were accompanied with sad mood in 12 (17.4%), lack of pleasure in 10 (14.5%), disturbed sleep in 21 (30.4%), fatigue in 8 (11.6%), crying spells in 7 (10.1%), forgetfulness in 8 (11.6%), free floating anxiety in 17 (24.6%), palpitations in 10 (14.5%), fear of crowds in 8 (11.6%), nausea and vomiting in 5 (7.2%), non-specific aches and pains in 10 (14.5%).

**Table 3:** Presenting complaints

S. No.	Presenting complaints	*n*	(%)
1.	Sad mood	12	(17.4)
2.	Lack of pleasure	10	(14.5)
3.	Disturbed sleep	21	(30.4)
4.	Fatigue	8	(11.6)
5.	Crying spells	7	(10.1)
6.	Forgetfulness	8	(11.6)
7.	Free floating anxiety	17	(24.6)
8.	Palpitation	10	(14.5)
9.	Fear of crowds	8	(11.6)
10.	Nausea and vomiting	5	(7.2)
11.	Body ache	10	(14.5)

## DISCUSSION

It was not until the end of the nineteenth century that Freud categorically associated the concepts of psychopathology with commonplace migraine. Wolff has been credited with developing the influential notion of ‘the migraine personality’ that he characterized as a medley of ‘personality features and reactions dominant in individuals with migraine’, including ‘feelings of insecurity with tension manifested as inflexibility, conscientiousness, meticulousness, perfectionism, and resent-ment’.^9^

Numerous epidemiological studies have revealed that psychiatric disorders (e.g. depression and anxiety) occur with greater frequency among recurrent headache patients than among the general population.^5^,^6^ Sixty-nine out of 80 subjects (86.25%) with complaints of headache had psychiatric morbidity according to the DSM-IV classification in this study. Among these 50 were men (72.5%). A large number of women patients, especially middle-aged ones, had migraine as well as depression. Longitudinal data indicate that relative to men, women are four-times more likely to develop migraine and two-times more likely to develop major depression.^9^

Fifty subjects (72.5%) were between 20 and 60 years of age. Most of the subjects with migraine had onset during teenage or early twenties while those with tension headache had a middle age onset; they were mostly women. Sixty subjects (86.5%) were earning less than Rs 2000 and were more susceptible to onset of headache as well as psychiatric morbidity.

Eighteen subjects (26%) were illiterate, 23 (33.3%) were unmarried and 6 persons (9%) had lost a spouse; 26 (38%) had a nuclear family, 19 (28%) were unemployed. Family history of mental illness and past history of mental illness was present in 5 (7%) and 2 (3%) subjects, respectively. They were all susceptible to headache and depression.

The comorbidity reported by Alvin *et al.*^9^ is as follows: MDD (34%), dysthymia (9%), bipolar II (4%), manic episode (5%), panic disorder (11%), generalized anxiety disorder (GAD) (10%), obsessive–compulsive disorder (OCD) (9%), phobia (40%), illicit drug use (20%), and nicotine dependence (33%). In comparison, this study shows MDD (32%), dysthymia (13%), panic disorder with agoraphobia (14.5%), phobia (2.9%), somatoform disorder (6%), substance dependence (26%) including nicotine dependence. The incidence of MDD, dysthymia, panic disorder is comparable, but the results of this study report no GAD, OCD or bipolar disorder.^9^,^10^

Wacogne *et al.*^11^ measured the intensity of stress, anxiety and depression in a sample of 141 migraineurs compared with a control group of 109 non-migraine workers matched for age and sex. Their results indicated that stress and anxiety were higher in the migraine group than in the control group. The main symptoms were ‘morning fatigue’, ‘intrusive thoughts about work’, ‘feeling under pressure’, ‘impatience’, and ‘irritability’. In the present study, disturbed sleep (30%), free floating anxiety (25%), sad mood (17%), lack of pleasure (14.5%), body ache (14.5%), fatigue (11.6%) were the main complaints.

Headache may be a form of ‘somatization’^12^ (a term used for the pathology, e.g. depression) when patients cannot verbalize their mental symptoms but present them by way of somatic symptoms. It was also called ‘depressive equivalent’ and was considered a typical manifestation of depression in non-industrialized countries.^13^,^14^ This hypothesis has been challenged by Patel^2^ who proved that this phenomenon is also common in industrialized countries. This may be a cross-cultural phenomenon. However, there is at least some evidence that headache can be a manifestation of a somatoform disorder.^15^ The most common somatoform disorder associated with headache was ‘undifferentiated somatoform disorder’. In somatoform disorder, headache would represent only one of many medically unexplained somatic complaints such as fatigue, loss of appetite, gastrointestinal symptoms, and urinary complaints.^10^

Recent characterizations of psychopathology and head-ache have implicated shared neuropathic mechanisms between migraine and affective disorders and bidirectional influences. Both concepts refer to neuroplastic processes in corticolimbic structures, where an expanding corticolimbic field becomes activated by both nociceptors and psychological stimuli over a period of time, resulting in an integrated relationship between migraine (or pain) and psychiatric disturbance in susceptible individuals.^9^

Evidence suggests that patients with elevated psycho-logical symptoms are more likely to seek medical assistance. When present, psychiatric comorbidity often complicates management of headache and portends a poorer prognosis for treatment of headache. These results indicate that patients with long history and high frequency of headaches might benefit from psychiatric evaluation.^16^ Physicians must be sensitized to look for psychiatric symptoms in patients presenting with headache.

## CONCLUSIONS

- Headache was the main somatic presentation of psychiatric morbidity in nearly 80% of subjects in this study.
- The associated psychiatric morbidity included depression, dysthmia, anxiety, somatoform disorder, phobia and substance abuse.
- Middle-aged women with migraine were more likely to have psychiatric morbidity.
- Also, people who were illiterate, unemployed, or had lost a spouse and with a family and past history of mental illness were more likely to develop mental illness.
- Disturbed sleep, free floating anxiety, sad mood, lack of pleasure, body ache and fatigue were the main presenting complaints along with headache.

## LIMITATIONS

- The authors were not able to use any specialized instru-ments to rate anxiety, depression, etc. due to paucity of time.
- The data were presented merely as percentages.
- Better planned, longitudinal studies are required to study this area further.
